# Global epidemiology and socioeconomic correlates of hypopharyngeal cancer in 2020 and its projection to 2040: findings from GLOBOCAN 2020

**DOI:** 10.3389/fonc.2024.1398063

**Published:** 2024-09-02

**Authors:** Seyed Ehsan Mousavi, Mehran Ilaghi, Yasaman Mirzazadeh, Alireza Mosavi Jarrahi, Seyed Aria Nejadghaderi

**Affiliations:** ^1^ Neurosciences Research Center, Aging Research Institute, Tabriz University of Medical Sciences, Tabriz, Iran; ^2^ Department of Community Medicine, Social Determinants of Health Research Center, Faculty of Medicine, Tabriz University of Medical Sciences, Tabriz, Iran; ^3^ Institute of Neuropharmacology, Kerman Neuroscience Research Center, Kerman University of Medical Sciences, Kerman, Iran; ^4^ Faculty of Medicine, Ardabil University of Medical Sciences, Ardabil, Iran; ^5^ Cancer Research Centre, Shahid Beheshti University of Medical Sciences, Tehran, Iran; ^6^ West Asia Organization for Cancer Prevention, Sabzevar, Iran; ^7^ HIV/STI Surveillance Research Center, and WHO Collaborating Center for HIV Surveillance, Institute for Futures Studies in Health, Kerman University of Medical Sciences, Kerman, Iran; ^8^ Systematic Review and Meta−analysis Expert Group (SRMEG), Universal Scientific Education and Research Network (USERN), Tehran, Iran

**Keywords:** hypopharyngeal neoplasm, epidemiology, incidence, mortality, GLOBOCAN

## Abstract

**Background:**

Hypopharyngeal cancer (HC) comprises less than 5% of all malignant tumors in the head and neck. They often present at an advanced stage, thereby resulting in high mortalities. We aimed to report the epidemiology of HC globally, regionally, and nationally by age, sex, and socioeconomic status in 2020 and its projection to 2040.

**Methods:**

Data on HC incidence and mortality were extracted from the GLOBOCAN 2020. Age-standardized incidence rate (ASIR), age-standardized mortality rate (ASMR), and mortality-to-incidence ratios (MIRs) were calculated. We used bivariate correlation test, presenting results through Pearson’s correlation coefficient (r) to investigate the correlation between the metrics, human development index (HDI), and current healthcare expenditure (CHE) as a percentage of gross domestic product (GDP) (CHE/GDP).

**Results:**

In 2020, there were 84254 new HC cases globally (ASIR: 0.91 per 100000). Moreover, HC resulted in 38599 mortalities in 2020 (ASMR: 0.41). Furthermore, the global MIR of HC was 0.45. The ASIR and ASMR of HC were higher in men than women. Also, HDI demonstrated significant correlations with HC ASIR (r= 0.249, p<0.01), ASMR (r= 0.185, p<0.05), and MIR (r= -0.449, p<0.001). Moreover, a weak significant correlation was also observed between CHE/GDP and MIR (r= -0.295, p<0.001). The number of new HC cases and mortalities were estimated to increase by 50% and 55% in 2040, respectively.

**Conclusion:**

HC is a relatively rare cancer but with a substantial sex and geographic divide in distribution. Key priorities should thus include establishing high-quality cancer registries worldwide.

## Introduction

The hypopharynx, also referred to as the laryngopharynx, is the most terminal part of the pharynx, functioning as a crucial pathway for digestion and respiration (1). Anatomically extending from the tip of the epiglottis superiorly, continuing to the lower border of the cricoid cartilage (2). The pyriform sinus is the primary source for the majority of tumors (70%) in this region, while approximately 25% emerge in the posterior pharyngeal wall, and the remaining mostly develop in the post-cricoid region (3).

Most hypopharyngeal cancers (HCs) originate from the epithelial layer of the mucous membrane covering the upper aero-digestive tract, predominantly squamous cell carcinomas. Less common types of cancers arising from the hypopharynx include basaloid squamoid carcinomas, spindle cell carcinomas, small cell carcinomas, undifferentiated carcinomas, and carcinomas of the minor salivary glands, which constitute less than 5% of all HCs (4). Several risk factors, including gastroesophageal reflux disease, genetic syndromes (e.g., Fanconi anemia and Plummer-Vinson syndrome), workplace exposures, and race have been reported for HC (5–8).

In 2020, HC accounted for about 85,000 incident cases and above 38,000 mortalities worldwide, which represents 0.4% of incidents and mortalities of all cancers globally (9). The prevalence of HC tends to increase notably among individuals aged 70 and older and is greater among males (9–11). Regardless of the low incidence of HC, that is <5% of all head and neck cancer, it is often presented at an advanced stage with poor prognosis (12–14). Based on the cancer stage, age, and the treatment plan, the survival rates may differ. In the United States, the predicted 5-year relative survival rate for early localized stage of this cancer has been reported to be 61%, with reduced rates to 28% in cases of distant metastasis (6).

Previous studies have reported the epidemiology of HC in several countries like the United States (15), Denmark (13), and the Netherlands (16). Also, the capstone paper of Global Cancer Observatory (GLOBOCAN) has reported the epidemiology of 36 cancers globally (9). However, the abovementioned studies were not focused on the global epidemiology of HC specifically. Therefore, we aimed to evaluate incidence and mortality of the HC at the global, regional, and national levels, by age, sex, and socioeconomic status in 2020. Also, we estimated the projection of the cancer to 2040 using the last version of the GLOBOCAN data.

## Methods

### Data sources

Epidemiological data on HC (International Classification of Disease-10: C12–13), were obtained from GLOBOCAN, which is a public access database maintained by the International Agency for Research on Cancer and the World Health Organization (WHO). This database provided contemporary estimates of cancer epidemiology for 36 major types of cancer classified by sex and age for 185 countries and 30 world regions (9). The data sources and hierarchy of methods have been described in detail in the previous studies (9, 17). In brief, the observed national incidence and mortality rates were forecasted for 2020, the recent incidence and mortality rates were applied to the 2020 population, rates were obtained from the national mortality data via modeling using mortality-to-incidence ratios (MIRs) from cancer registries in the country, rates were calculated from national incidence or mortality estimates via modeling using MIRs obtained from cancer registries in neighboring countries, the age- and sex-specific national incidence rates for all malignancies were calculated by averaging overall rates from neighboring countries, rates were calculated as the average of those from selected neighboring nations (17).

Population data for 2020 were extracted from the 2019 revision of the World Population Prospects of United Nations. The Human Development Index (HDI) data were extracted from the Human Development Report Office of the United Nations Development Programme (7). Indicators for current healthcare expenditure (CHE) as a percentage of gross domestic product (GDP) for the year 2019 were extracted from the Global Health Observatory data repository of WHO (18). This study followed the Guidelines for Accurate and Transparent Health Estimates Reporting (GATHER) statement (19) as well as the Strengthening the Reporting of Observational Studies in Epidemiology (STROBE) statement (20).

### Study variables

We gathered and reported two summary measures of HC including incidence and mortality and used them to calculate MIRs. The MIR is a measure of healthcare quality, in which a low value is considered as superior cancer care in terms of screening, therapy, and overall disease management (21). Also, we reported the cumulative risk percentage of incidence and mortality of HC to describe the risk of developing and dying from HC before the age of 75 years. Another variable in this study was CHE as a percentage of GDP (CHE/GDP%). The CHE/GDP% indicator is used to assess the allocation of financial resources to the health sector within a nation (18).

We utilized the HDI for providing a summary measure to account for the socioeconomic status of countries based on fundamental areas of human development, which are life expectancy at birth, the average of education attained by people aged 25 years and older, the projected number of years of education for children of school age, and gross national income per capita in purchasing power parity (in US dollars) (7). HDI is categorized into four quartiles which are low, medium, high, and very high. However, it should be noted that this categorization does not apply to China and India, as per the GLOBOCAN approach (22). Furthermore, correlation tests were conducted using HDI point values for each nation, with the exception of 10 countries/territories for which HDI estimates were not available.

In brief, in this study the results were stratified by sex, age group, six WHO regions (Africa, Americas, Eastern Mediterranean, Europe, Southeast Asia, and Western Pacific), six continents (Africa, Asia, Europe, Latin America and the Caribbean, North America, and Oceania), World Bank (WB) income levels, and HDI.

### Statistical analysis

We provided tables and figures displaying the number of new cases and mortalities, crude incidence and mortality rates, age-standardized incidence rate (ASIR), and age-standardized mortality rate (ASMR). All rates were expressed per 100000 population. The direct standardization method was used to calculate age-standardized rates with the Segi-Doll World standard population from 1966. Standardization is critical when comparing diverse populations with varying age distributions. Age groups were categorized at 10-year intervals (0–9, 10–19, 20–29, 30–39, 40–49, 50–59, 60–69, and 70+). The relationship between HC incidence rate, mortality rate, and estimated MIR with HDI and CHE/GDP% was analyzed for countries/territories with available data. This analysis was done using the bivariate correlation test, and the results were reported using the Pearson’s correlation coefficient. The coefficient was categorized into three ranges based on its absolute value which were strong (>0.5), moderate (0.5–0.3), and weak (<0.3). A p-value of less than 0.05 from a two-sided test was considered as statistically significant.

The projected number of new cancer cases or mortalities in a nation or area between 2025 and 2040 was calculated by multiplying the age-specific incidence or mortality rates, calculated for 2020, by the corresponding expected population for the years 2025 to 2040. Data cleaning, analysis, and visualization were carried out in R statistical software, version 4.3.2 (23).

## Results

### Global, regional, and national HC incidence and mortality

In 2020, there were a total number of 84254 (95% uncertainty interval (UI): 76654.6–92606.8) new HC cases globally, representing a crude rate of 1.10 per 100000, an ASIR of 0.91 per 100000, and an all-age cumulative risk of 0.18%. Moreover, HC resulted in 38599 (95% UI: 34237.5–43516.2) mortalities in 2020, corresponding to a crude rate of 0.50, an ASMR of 0.41, and a cumulative risk of 0.09% (**Table 1**).

**Table 1:** Hypopharyngeal cancer incidence and mortality metrics in 2020 for different geographic and socioeconomic categories.

Location	Incidence					Mortality					MIR
	Number	Uncertainty interval	Crude rate	ASIR	Cumulative risk (%)	Number	Uncertainty interval	Crude rate	ASMR	Cumulative risk (%)	
**World**	84254	76654.6-92606.8	1.10	0.91	0.18	38599	34237.5-43516.2	0.50	0.41	0.09	0.45
WHO regions											
**WHO Africa (AFRO)**	1522	704.8-3286.7	0.14	0.25	0.05	1119	464.8-2693.8	0.10	0.18	0.04	0.71
**WHO Americas (PAHO)**	4892	4369.9-5476.5	0.48	0.34	0.07	1740	1462.5-2070.2	0.17	0.12	0.03	0.35
**WHO East Mediterranean (EMRO)**	3022	2027.4-4504.6	0.41	0.54	0.11	1405	917.9-2150.5	0.19	0.25	0.06	0.46
**WHO Europe (EURO)**	19730	17323.8-22470.4	2.10	1.30	0.21	9721	8216.6-11500.8	1.00	0.61	0.11	0.48
**WHO South-East Asia (SEARO)**	38948	35915.0-42237.1	1.90	1.90	0.41	16336	12709.4-20997.5	0.81	0.80	0.19	0.43
**WHO Western Pacific (WPRO)**	16113	11214.9-23150.3	0.82	0.52	0.12	8268	4755.2-14375.9	0.42	0.26	0.07	0.51
Continent											
**Africa**	2065	1050.8-4058.1	0.15	0.26	0.06	1439	667.4-3102.8	0.11	0.18	0.04	0.73
**Asia**	58058	52960.7-63645.9	1.30	1.00	0.22	25906	21450.3-31287.2	0.56	0.46	0.11	0.43
**Europe**	18996	17891.1-20169.1	2.50	1.40	0.23	9418	8483.0-10456.0	1.30	0.68	0.12	0.52
**Latin America and the Caribbean**	2430	1977.1-2986.6	0.37	0.31	0.07	1076	936.2-1236.6	0.16	0.14	0.03	0.43
**Northern America**	2462	2355.6-2573.1	0.67	0.37	0.07	664	606.9-726.5	0.18	0.09	0.02	0.27
**Oceania**	243	197.8-298.5	0.57	0.38	0.09	96	67.0-137.7	0.22	0.14	0.04	0.39
World Bank income levels											
**Low income**	859	510.3-1446.0	0.14	0.25	0.05	583	325.7-1043.6	0.10	0.17	0.04	0.71
**Low middle income**	45678	41444.4-50344.0	1.50	1.70	0.37	20004	15180.4-26360.4	0.66	0.76	0.18	0.44
**Upper middle income**	15751	14186.9-17487.5	0.54	0.39	0.08	8204	6941.5-9696.1	0.28	0.20	0.04	0.52
**High income**	21957	20692.9-23298.3	1.80	0.97	0.18	9807	8966.0-10726.9	0.80	0.40	0.09	0.44
HDI categories											
**Low HDI**	1117	549.9-2269.0	0.11	0.21	0.05	813	368.7-1792.8	0.08	0.16	0.04	0.73
**Medium HDI**	42745	39166.3-46650.6	1.80	2.00	0.44	18296	13428.4-24928.0	0.79	0.87	0.21	0.44
**High HDI**	13024	11444.1-14822.0	0.45	0.33	0.07	6830	5791.1-8055.3	0.23	0.17	0.04	0.51
**Very high HDI**	27359	25832.0-28976.2	1.70	1.00	0.18	12659	11464.1-13978.5	0.81	0.44	0.09	0.48

ASIR, Age-standardized incidence rate; ASMR, Age-standardized mortality rate; MIR, Mortality-to-incidence ratio; WHO, World Health Organization; HDI, Human Development Index. Rates are presented per 100000 population.

The WHO regions with the highest HC ASIRs were Southeast Asia (1.90) and Europe (1.30), while Africa (0.25) and the Americas (0.34) accounted for the lowest ASIRs. Similarly, the highest ASMRs were observed in the WHO regions of Southeast Asia (0.80) and Europe (0.61), while those with the lowest ASMRs were the Americas (0.12) and Africa (0.18) (**Table 1**).

Continents with the highest HC ASIRs were Europe (1.40) and Asia (1.00), while Africa (0.26) was the continent with the lowest ASIR. Furthermore, Europe (0.68) and Asia (0.46) remained the continents with the highest AMSRs, while Northern America (0.09) accounted for the least ASMR among continents (**Table 1**).

Analyzing the rates according to the WB income levels demonstrated that the low-middle income quartile accounted for the highest HC ASIR (1.70) and ASMR (0.76). Based on the HDI categories, both the highest ASIR (2.00) and ASMR (0.87) were observed in the medium HDI category (2.00) (**Table 1**).

**Figure 1:**
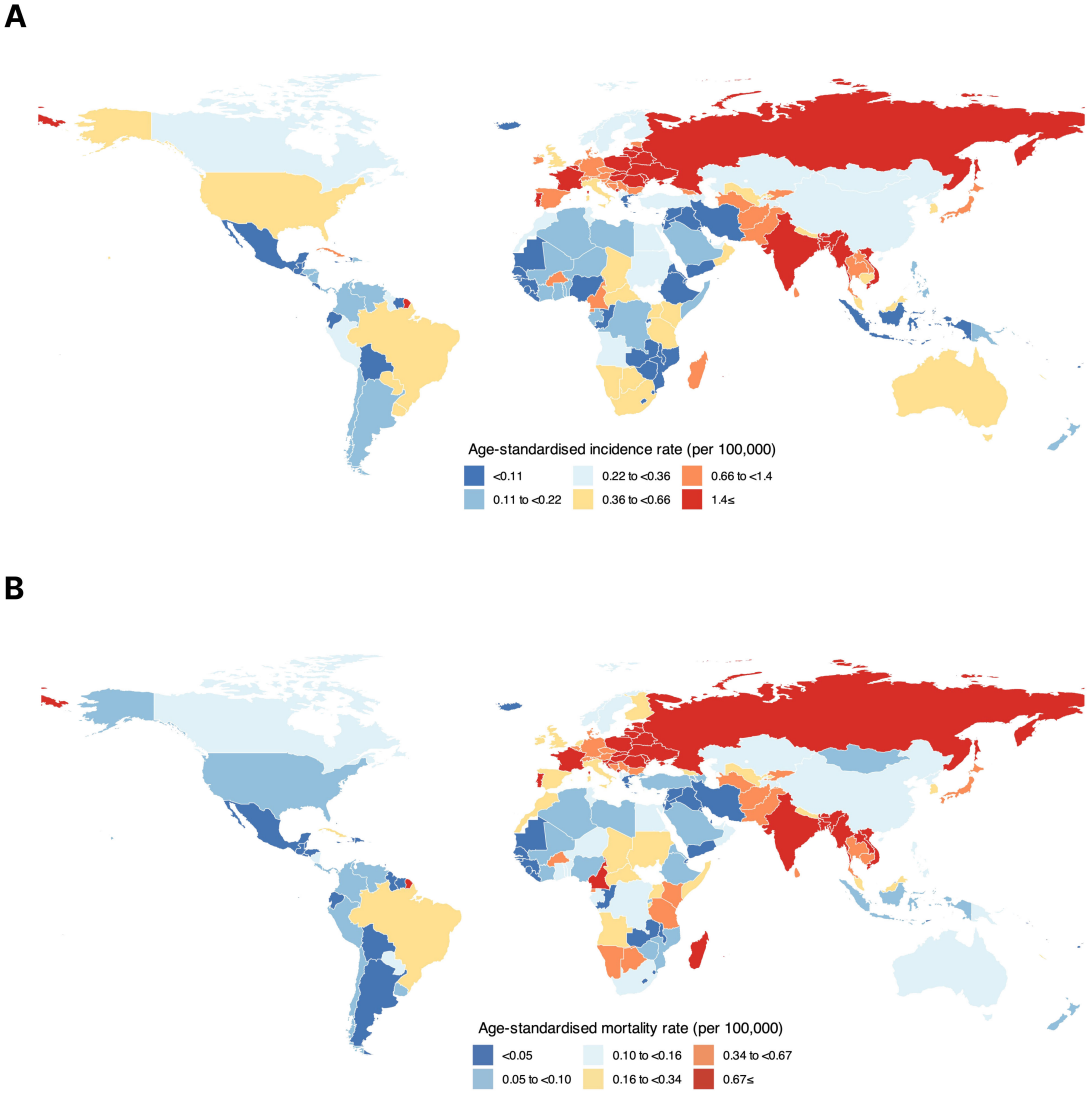
Global distribution of hypopharyngeal cancer based on **(A)** age-standardized incidence rate and **(B)** age-standardized mortality rate among both sexes combined.

Among all countries, HC ASIRs were the highest in Bangladesh (5.20), Hungary (3.10), and France (2.50) among both sexes combined. The country with the lowest HC ASIR was Zambia (0.01) (**Figure 1A**). Furthermore, the highest HC ASMRs were in Bangladesh (2.20), Hungary (2.10), and Slovakia (1.80) among both sexes combined, while the lowest ASMRs were in Zambia (0.01) and Jordan (0.01) (**Figure 1B**). The corresponding data for the national HC incidence and mortality metrics are provided in the **Supplementary File 1**.

### Global and regional MIR of HC

The global calculated MIR of HC in 2020 among both sexes combined was 0.45, with the highest ratio in the WHO region of Africa (0.71) and the lowest in the WHO region of the Americas (0.35). Among continents, Africa (0.73) accounted for the highest MIR, while Northern America (0.27) ranked at the bottom. According to the WB income categories, low-income countries had the highest MIR (0.71), which was followed by upper-middle-income (0.52), low-middle-income (0.44), and high-income (0.44) categories. Based on the HDI categories, low HDI countries (0.73) represented the highest MIR, followed by high HDI (0.51), very high HDI (0.48), and medium HDI (0.44) (**Table 1**).

### HC incidence and mortality in age groups

Globally, the 70+ age group accounted for the highest HC crude incidence (4.90) and mortality rates (2.80). Accordingly, the risk of developing HC before the age of 70 was 0.05%, and the risk of dying from HC before the age of 70 was 0.02%. Moreover, the 70+ age group had the highest MIR for HC (0.57). Excluding those aged 0–19 years, the lowest MIR was in the 50–59 group (0.38), followed by the 20–29 (0.40) and the 40–49 (0.40) age groups (**Table 2**).

**Table 2:** Global hypopharyngeal cancer incidence and mortality metrics for all age groups among both sexes combined.

Age group	Incidence			Mortality			MIR
	Number	Crude rate	Cumulative risk (%)	Number	Crude rate	Cumulative risk (%)	
0 to 9	49	0	0	18	0	0	0
10 to 19	109	0.01	0	61	0	0	0
20 to 29	552	0.05	0	291	0.02	0	0.40
30 to 39	2730	0.24	0	1457	0.13	0	0.54
40 to 49	9896	1.00	0.01	3883	0.40	0	0.40
50 to 59	21768	2.60	0.03	8171	0.98	0.01	0.38
60 to 69	26721	4.50	0.05	11808	2.00	0.02	0.44
70+	22429	4.90	0.09	12910	2.80	0.06	0.57

MIR, Mortality-to-incidence ratio.

### HC incidence and mortality among sex groups

#### Men

Among men, there were a total of 70254 (95% UI: 63472.7–77759.9) new HC cases globally in 2020, corresponding to a crude rate of 1.80 per 100000, an ASIR of 1.60, and an all-age cumulative risk of 0.33%. The total number of HC mortalities in men was 32303 (95% UI: 28352.2–36804.3) in 2020, representing a crude rate of 0.82, an ASMR of 0.72, and a cumulative risk of 0.17%. The global HC MIR for men was 0.46 (**Supplementary Table 1**).

**Figure 2:**
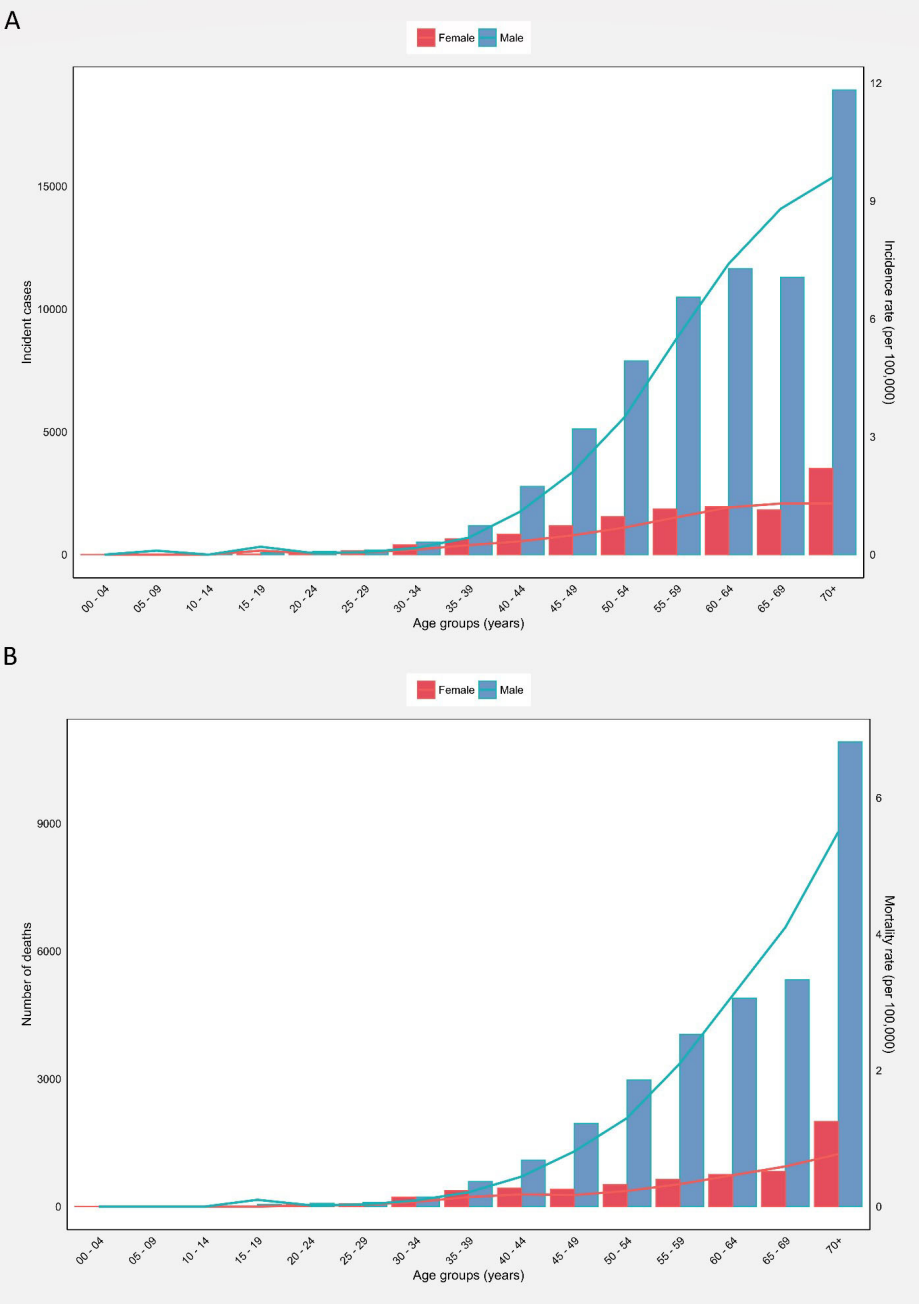
**(A)** Global number of incident cases and incidence rate, and **(B)** Global number of mortalities and mortality rate of hypopharyngeal cancer among males and females in each age group.

The 70+ age group accounted for the highest HC crude incidence rate (9.60), mortality rate (5.50), and MIR (0.57) among men (**Supplementary Table 2**). Accordingly, there were minimal variations in the incidence and mortality rates of HC among men till 30–34 age groups, with a substantial increase afterwards in both incidence (**Figure 2A**) and mortality (**Figure 2B**) rates peaking at the 70+ age group. The incidence and mortality numbers and rates were generally higher in men than women.

Among all countries, Bangladesh (8.60), Hungary (5.90), and Belarus (5.30) represented the highest HC ASIRs in men (**Supplementary Figure 1**). The countries with the highest HC ASMRs among men were Hungary (4.10), Bangladesh (3.70), Belarus (3.70), and Slovakia (3.70) (**Supplementary Figure 2**, **Supplementary File 1**).

#### Women

Among women, a total number of 14000 (95% UI: 10847.2–18069.2) new HC cases were reported globally in 2020, corresponding to a crude rate of 0.36 per 100000, and ASIR of 0.29, and an all-age cumulative risk of 0.05%. Moreover, the total number of HC mortalities in women was 6296 (95% UI: 4653.8–8517.7) in 2020, accounting for a crude rate of 0.16, an ASMR of 0.13, and a cumulative risk of 0.03%. The global HC MIR for women was 0.44 (**Supplementary Table 3**).

The 70+ age group represented the highest HC crude incidence (1.30) and mortality rates (0.77). The highest MIRs were observed in the 30–39 (0.61) and 70+ (0.59) age groups (**Supplementary Table 4**). Accordingly, there were minimal fluctuations in the incidence and mortality rates of women till the 30–34 age group. Afterward, both incidence (**Figure 2A**) and mortality (**Figure 2B**) rates witnessed an increasing trend, peaking at 70+ age. The countries with the highest HC ASIRs among women were Bangladesh (1.50), Bhutan (1.30), and Burkina Faso (1.10) (**Supplementary Figure 3**). Moreover, the countries with the highest HC ASMRs among women were Burkina Faso (0.96), Bhutan (0.69), and Bangladesh (0.66) (**Supplementary Figure 4**, **Supplementary File 1**).

### Correlation between HC incidence, mortality, MIR, HDI, and CHE/GDP

**Figure 3:**
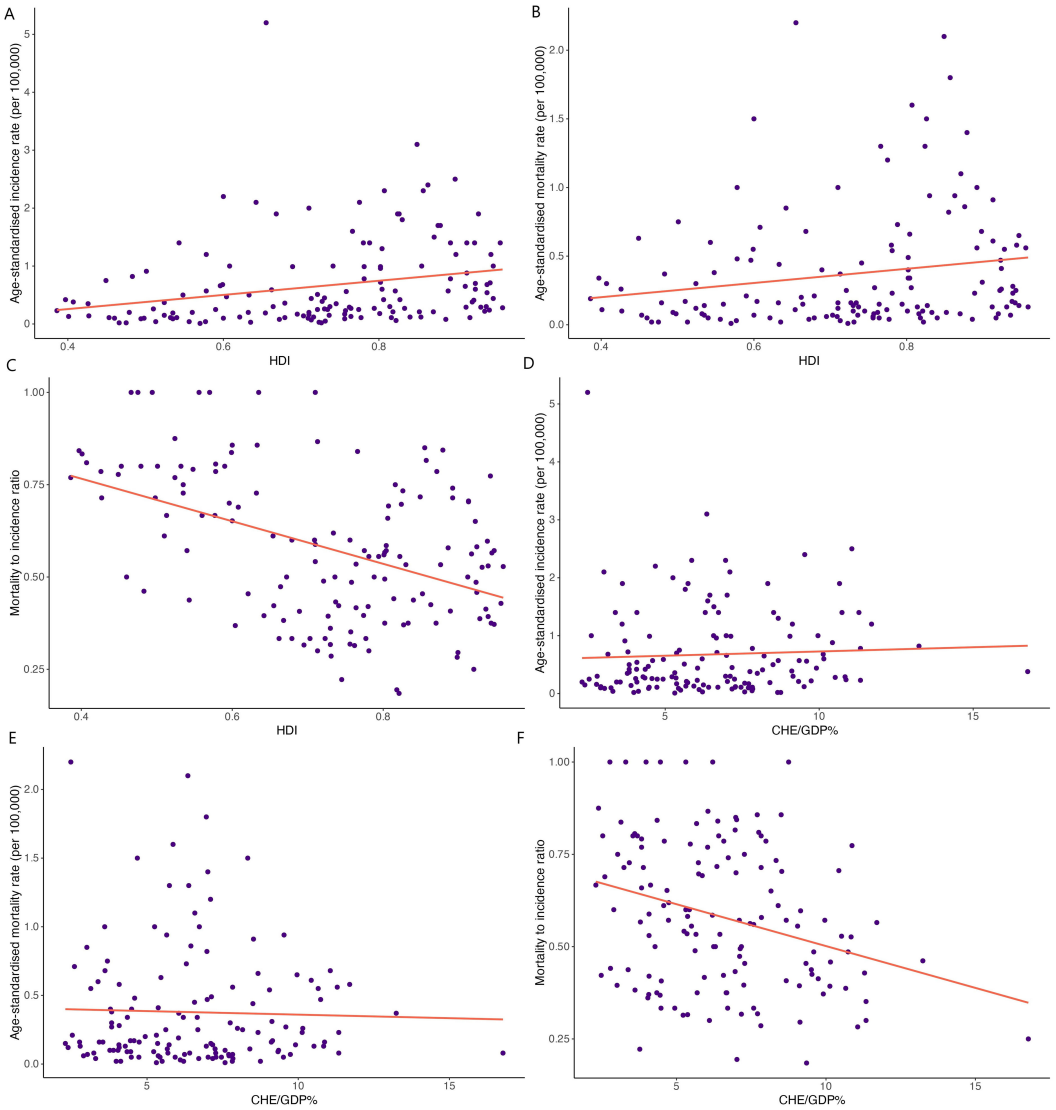
Correlations between human development index (HDI) and **(A)** age-standardized incidence rate, **(B)** age-standardized mortality rate, and **(C)** mortality-to-incidence ratio. Correlations between the current healthcare expenditure to gross domestic product (CHE/GDP%) and **(D)** age-standardized incidence rate, **(E)** age-standardized mortality rate, and **(F)** mortality-to-incidence ratio.

HDI demonstrated significant correlations with HC ASIR (correlation coefficient= 0.249, p<0.01; **Figure 3A**), ASMR (correlation coefficient= 0.185, p<0.05; **Figure 3B**), and MIR (correlation coefficient= -0.449, p<0.001; **Figure 3C**). Moreover, a weak significant correlation was observed between CHE/GDP and MIR (correlation coefficient= -0.295, p<0.001; **Figure 3F**). However, CHE/GDP did not show significant correlations with HC ASIR (p= 0.55) (**Figure 3D**) and ASMR (p= 0.72) (**Figure 3E**).

### Projections of HC incidence and mortality to 2040

**Figure 4:**
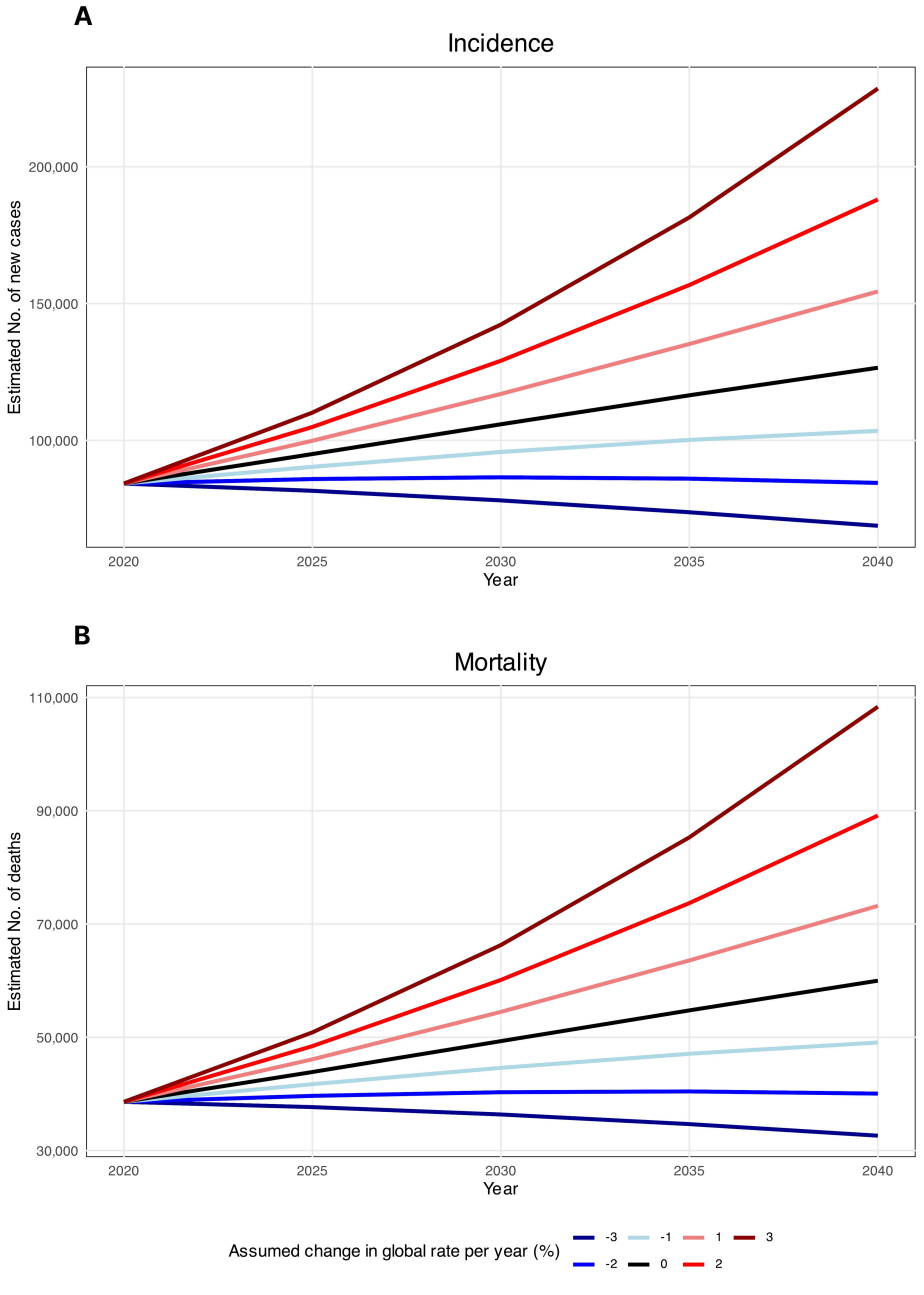
Estimated hypopharyngeal cancer **(A)** incidence and **(B)** mortality from 2020 to 2040. The baseline scenario (represented by the black line), posits that there are no alterations in incidence and mortality, meaning that any rise in numbers is solely attributed to changes in population size and composition. Due to the unlikelihood of stable incidence rates, alternative scenarios are provided.

The estimated number of new HC cases was determined to increase by 50%, from 84254 cases in 2020, to 126000 cases in 2040 (**Figure 4A**). Likewise, HC mortalities were anticipated to surge by 55%, from 38599 mortalities in 2020 to 60000 mortalities in 2040, assuming that the rates in 2020 remained constant (**Figure 4B**). These projections were computed solely by considering changes in the global population size and age structure without factoring in potential changes in age-specific incidence rates either globally or within individual countries. Decreases in global incidence and mortality rates would need to be greater than 2.1% and 2.2%, respectively, to ensure there would be fewer HC cases in 2040 than there were in 2020.

## Discussion

Utilizing data from GLOBOCAN 2020, this study provided a comprehensive assessment of the contemporary global, regional, and national epidemiology of HC in 2020. We also assessed the disparities in cancer care and the potential contribution of development level and healthcare expenditure on HC incidence and mortality metrics. Overall, incidence and mortality rates demonstrated substantive geographic variations, with the highest ASIRs and ASMRs noted in Southeast Asia and Europe WHO regions. At the national level, countries such as Bangladesh, Hungary, and France had the highest incidence rates, while Bangladesh and Hungary remained among the countries with the highest mortality. Furthermore, the incidence and mortality of HC was disproportionately higher among men. Intriguingly, we found positive, albeit weak, correlations between HDI and incidence metrics, suggesting that both incidence and mortality rates are higher in developed regions. Importantly, our estimates showed that assuming constant rates, the absolute HC burden is expected to increase by 2040, emphasizing the need for reinforcement of preventive strategies.

HC is generally considered a relatively rare cancer entity. The global incidence and mortality estimates in our study for 2020 are mostly consistent with prior assessments of the HC burden in 2018 (24). Although reports on global temporal patterns of HC are scarce, several previous studies have reported the incidence trends at a national level. Decreasing incidence with an average annual percent change of −2.0% has been reported according to a United States population-based study between 1973 and 2010 (25). Similar decreasing pattern in incidence rates (from 1.0 to 0.8 per 100000 population) has been reported in another study during 1974–1999 in the United States (26). However, a Danish nationwide study has reported increasing ASIRs of HC from 0.3 to 1.1 per 100000 population from 1980 to 2014, representing an increase of 4.1% per year (13). The same increasing patterns have been reported in Germany during 1996–2005 (27), and in the Netherlands between 1989 and 2013 (16). The decreasing incidence rates in the US, in contrast to increasing rates observed in several European countries, align with our findings showing that northern America has lower incidence rates as compared to Europe, though global studies on incidence trends are warranted to determine the global temporal patterns of HC.

Our findings showed that Southeast Asia and Europe had the highest ASIRs in 2020. In terms of the epidemiology of esophageal cancer, Eastern Asia had the highest incidence rates in both males and females, and Northern Europe were among regions with a high ranks of incidence rates in 2020 (28). When discussing the incidence rates of HC, it is essential to consider the frequent co-occurrence cancers. Studies have shown that an almost significant proportion of patients with HC are also diagnosed with esophageal squamous cell carcinoma (ESCC) (29, 30). For instance, the article by Huang et al. found that 45.5% of patients with HC had concurrent ESCC (31). This suggests a potential etiological link or shared risk factors between the two malignancies, such as tobacco and alcohol use (30). The lowest ASIRs were observed in Africa and the Americas in 2020, according to our findings. One of the factors that may contribute to the lower ASIRs of HC observed in these regions is the absence of widespread screening systems for upper gastrointestinal cancers. In contrast, the majority of HC cases in Asia and Europe are detected through upper gastrointestinal endoscopy. These procedures are more routinely performed in these regions due to the higher prevalence of upper gastrointestinal cancers and established screening programs. The implementation of such screening systems in Asia and Europe likely facilitates the early detection and diagnosis of HC, leading to higher reported incidence rates (32). Therefore, the lack of similar screening programs in North America and Africa may result in underreporting and later-stage diagnoses, contributing to the observed differences in incidence rates. This discrepancy underscores the need for increased screening efforts and awareness in regions with lower reported incidence rates (33). Regarding the mortality rates, it is noted that the ASMR and MIRs for HC may be underestimated due to the high mortality from esophageal cancer in these patients. Many individuals diagnosed with HC die from esophageal cancer before the HC itself becomes fatal. This is because esophageal cancer often develops prior to HC in the majority of cases. In this regard, patients with both HC and esophageal cancer have a poor prognosis than those without esophageal cancer (34).

The sex and age patterns discerned in HC incidence align with established evidence pointing toward substantially higher occurrence among older men (16, 25). This likely reflects cumulated exposure to key risk factors such as smoking and alcohol over the lifespan among men. The peak in older ages is indicative of the fact that HC is among the cancers which is typically diagnosed at later ages, thereby the high stage at diagnosis results in reduced overall survival. Individuals suffering from hypopharyngeal tumors may remain asymptomatic until there is infiltration of the larynx or the onset of nodal metastasis. The hypopharynx possesses a rich lymphatic network, facilitating the early spread of tumor to the nodal basins of the neck and elevates the probability of distant metastasis (10, 35). Delayed diagnosis contributes to a poor prognosis for HC, rendering it one of the most unfavorable prognoses among all head and neck cancers with a reported 5-year overall survival rate of 30–35% (36).

The findings of our study suggest that clear geographical disparities seem to exist in HC incidence, mortality, and quality of care. Although Africa represented the lowest ASIR according to our analysis, the MIR values in this region were the highest when compared to other continents, possibly highlighting the lowest quality of care and cancer management in this continent. On the other hand, while Europe demonstrated the highest ASIR among all continents, the MIR values were relatively lower compared to Africa. To gain a better insight into the correlative factors of incidence rates with nations’ development, we also assessed the correlation of incidence metrics with HDI and CHE/GDP. In health studies and population-based investigations, HDI is extensively employed as a composite indicator encompassing life expectancy, education, and income levels of individuals (37). Therefore, investigations of epidemiological metrics related to HC, in conjunction with the HDI as a socioeconomic measure and CHE/GDP serving as a financial indicator of health systems, elucidates potential disparities in HC diagnosis and the quality of care across the world. We observed that the countries with low HDI and low WB income levels represented the lowest ASIRs, but the highest MIR values. The low ASIR among less developed countries might generally suggest an impaired screening process, which might result in the underdiagnosis of cancer patients in these countries. However, the higher MIR discerned in less developed regions point to deficiencies in early diagnosis and access to optimal treatment, and generally a poorer quality of care. The utility of MIR as an indicator of cancer screening and care has been previously demonstrated in other cancers (21, 38), generally suggesting that less developed countries without robust screening programs and with limited access to health services demonstrate a higher proportion of mortalities among cancer cases. Therefore, improving health systems capacity for timely and accurate diagnostics paired with expanding coverage of evidence-based multimodality care for HC will be instrumental in bridging these divides.

On the other hand, analyzing the general correlations of developmental metrics with incidence rates, suggested a positive correlation between HDI and ASIR and ASMR. Accordingly, as described previously, the finding that HDI is positively correlated with ASIR is suggestive of more efficient screening processes in developed countries. Countries with higher HDI generally have better access to health services, including cancer screening and diagnostic tests, which could lead to more cases being detected at an early stage, thereby inflating the incidence rates. Moreover, populations in higher HDI countries generally have longer life expectancy with lower burdens of communicable diseases (39), therefore, cancers that predominantly affect older ages (like HC) are more likely to develop and be diagnosed. On the other hand, health behaviors, including smoking, alcohol, and high-fat diet might also be higher in more socioeconomically advantaged areas (40–43), which could potentially contribute to higher incidence rates reported in more developed regions. Our findings showed that the low HDI countries had the highest MIR values for HC, and we observed a generally negative correlation between MIR and both metrics of HDI and CHE/GDP.

Therefore, this finding could potentially reflect that the absolute disparities in mortality outcomes are most substantial between the low HDI and medium to very high HDI countries, resulting in exceptionally high MIR levels concentrated in the low HDI category. Moreover, these insights also highlight that while socioeconomic prosperity enables diagnostic improvements to drive detected incidence, concerted policy efforts are still needed to universally enable commensurate gains in cancer survival outcomes across all resource settings in order to alleviate global disparities.

Finally, the projections of the future HC burden offer concerning insights, as our estimates suggest a 50% rise in incident cases and a 55% increase in mortalities may occur by the year 2040, assuming rates remain constant at the 2020 levels. This translates to over 40,000 additional new annual cases and over 20,000 more mortalities expected globally after merely two decades. Moreover, the results indicate that decreases in incidence and mortality rates would need to outpace plausible declines expected from sole demographic shifts alone in order to reverse the mounting burden. Specifically, annual reductions exceeding 2.1% and 2.2% would be required in incidence and mortality, highlighting the need for accelerated progress at rates substantially higher than historically achieved. Overall, the stark projections provide an alarming outlook of the future burden but simultaneously offer a critical window of opportunity to reinforce evidence-based primary and secondary prevention policies targeting established risk factors as well as to improve early diagnosis and equitable access to quality care to mitigate the alarming trajectory expected based on current rates.

Our study has several strengths. First, it represents the most up-to-date analysis of HC burden leveraging data from GLOBOCAN 2020 to generate estimates at the global, regional, and national levels. Moreover, we utilized several metrics including MIR, HDI, and CHE/GDP to map the socioeconomic correlates and quality of care disparities across the world. Additionally, the long-term projections offer the first available estimates forecasting HC cases and mortalities to the year 2040. However, this study has certain limitations inherent to the GLOBOCAN methodology that warrant acknowledgment. The estimates are based on the best available country-level data but there remains variability in the completeness, accuracy, and representativeness of cancer registry coverage between nations. Misclassification and under-reporting are concerns, particularly in less developed regions without high-quality registries. Moreover, the exclusive focus on country-income and human development covariates might further preclude a comprehensive analysis of potential predictors shaping observed variations. Finally, the projected rise in HC cases and mortalities by 2040 in our analysis stems purely from anticipated population growth and shifts in age structure at the country level. We did not factor in potential changes over time in age-specific incidence and mortality rates within countries that could influence future burdens beyond simple demographic impacts. More precise prediction approaches such as age-period-cohort modeling could enable nuanced predictions. However, such high-quality longitudinal inputs remain scarce in most world regions presently. In addition, we did not have data on the epidemiology of esophageal cancer and its correlations with HC incidence or mortality. When reporting on the incidence or mortality rates of HC, it is difficult to ignore ESCC, because the majority of patients with HC have both. This co-occurrence of HC and esophageal cancer, as well as their effects of ASMRs and MIRs should be considered in future studies.

## Conclusions

HC represents a low-incidence cancer typically presenting in older ages, with a clear sex and geographical divide in distribution. Although low-developed countries represent the highest burden of cancer, the disparities in the quality of care seem to be less pronounced among countries with medium to very high HDI. Therefore, priority areas for health policy include establishing high-quality population-based cancer registries, monitoring of key risk factors, improving screening and early diagnosis, reducing treatment disparities, and strengthening health systems capacity, particularly with a focus on countries with low HDI.

## Data availability statement

The datasets presented in this study can be found in online repositories. The names of the repository/repositories and accession number(s) can be found below: https://gco.iarc.fr/today, United Nations Development Programme (https://hdr.undp.org/data-center/human-development-index#/indicies/HDI), and Global Health Observatory of World Health Organization [https://www.who.int/data/gho/data/indicators/indicator-details/GHO/current-health-expenditure-(che)-as-percentage-of-gross-domestic-product-(gdp)-(–)].

## Ethics statement

The present report was reviewed and approved by the Ethics Committee of Shahid Beheshti University of Medical Sciences, Tehran, Iran (ethics code: IR.SBMU.CRC.REC.1403.013).
