# Corrigendum: Global epidemiology and socioeconomic correlates of hypopharyngeal cancer in 2020 and its projection to 2040: findings from GLOBOCAN 2020

**DOI:** 10.3389/fonc.2024.1520064

**Published:** 2024-12-02

**Authors:** Seyed Ehsan Mousavi, Mehran Ilaghi, Yasaman Mirzazadeh, Alireza Mosavi Jarrahi, Seyed Aria Nejadghaderi

**Affiliations:** ^1^ Neurosciences Research Center, Aging Research Institute, Tabriz University of Medical Sciences, Tabriz, Iran; ^2^ Department of Community Medicine, Social Determinants of Health Research Center, Faculty of Medicine, Tabriz University of Medical Sciences, Tabriz, Iran; ^3^ Institute of Neuropharmacology, Kerman Neuroscience Research Center, Kerman University of Medical Sciences, Kerman, Iran; ^4^ Faculty of Medicine, Ardabil University of Medical Sciences, Ardabil, Iran; ^5^ Cancer Research Centre, Shahid Beheshti University of Medical Sciences, Tehran, Iran; ^6^ West Asia Organization for Cancer Prevention, Sabzevar, Iran; ^7^ HIV/STI Surveillance Research Center, and WHO Collaborating Center for HIV Surveillance, Institute for Futures Studies in Health, Kerman University of Medical Sciences, Kerman, Iran; ^8^ Systematic Review and Meta−analysis Expert Group (SRMEG), Universal Scientific Education and Research Network (USERN), Tehran, Iran

**Keywords:** hypopharyngeal neoplasm, epidemiology, incidence, mortality, GLOBOCAN

In the published article, there was an error in **Figure 3** as published. The mortality to incidence ratios in y-axis of panels C and F were not calculated correctly. The corrected **Figure 3** and its caption “**Figure 3**”. Correlations between human development index (HDI) and (A) age-standardized incidence rate, (B) age-standardized mortality rate, and (C) mortality-to-incidence ratio. Correlations between the current healthcare expenditure to gross domestic product (CHE/GDP%) and (D) age-standardized incidence rate, (E) age-standardized mortality rate, and (F) mortality-to-incidence ratio appear below.

**Figure 3:**
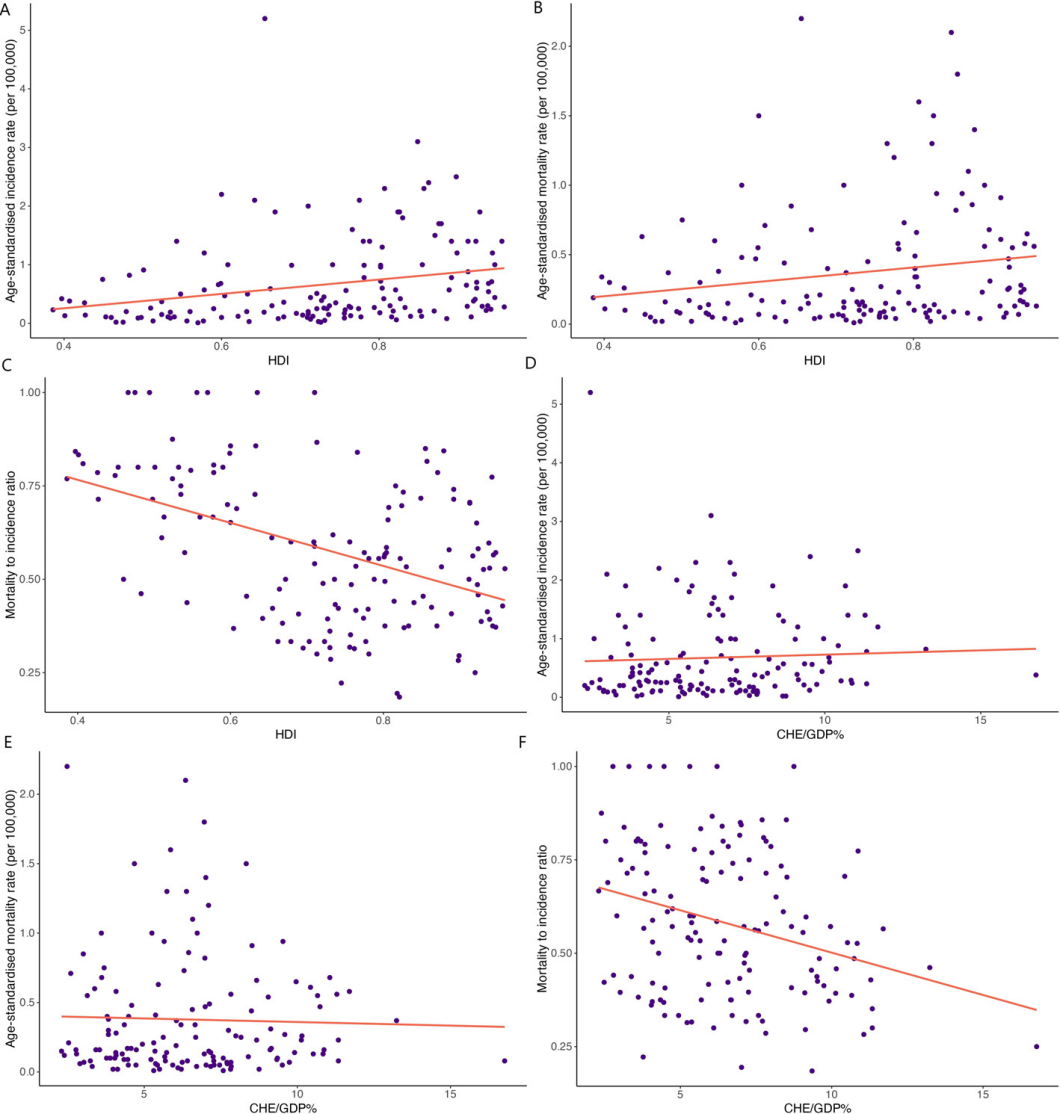
Correlations between human development index (HDI) and **(A)** age-standardized incidence rate, **(B)** age-standardized mortality rate, and **(C)** mortality-to-incidence ratio. Correlations between the current healthcare expenditure to gross domestic product (CHE/GDP%) and **(D)** age-standardized incidence rate, **(E)** age-standardized mortality rate, and **(F)** mortality-to-incidence ratio.

In the published article, there was an error in **Supplementary Data Sheet 2**. There was a mistake in the calculation of mortality to incidence ratios. The correct material statement appears below.

In the published article, there was an error. The values of correlation coefficient for the correlation between mortality to incidence ratios and human development index and current healthcare expenditure per gross domestic product were not correct.

A correction has been made to **Results**, *Correlation between HC incidence, mortality, MIR, HDI, and CHE/GDP*, Paragraph 1. This sentence previously stated:

“HDI demonstrated weak significant correlations with HC ASIR (correlation coefficient= 0.249, p<0.01; **Figure 3A**), ASMR (correlation coefficient= 0.185, p<0.05; **Figure 3B**), a moderate correlation with and MIR (correlation coefficient= 0.347, p<0.001; **Figure 3C**). Moreover, a weak significant correlation was observed between CHE/GDP and MIR (correlation coefficient= 0.279, p<0.001; **Figure 3F**).”

The corrected sentence appears below:

“HDI demonstrated significant correlations with HC ASIR (correlation coefficient= 0.249, p<0.01; **Figure 3A**), ASMR (correlation coefficient= 0.185, p<0.05; **Figure 3B**), and MIR (correlation coefficient= -0.449, p<0.001; **Figure 3C**). Moreover, a weak significant correlation was observed between CHE/GDP and MIR (correlation coefficient= -0.295, p<0.001; **Figure 3F**).”

In the published article, there was an error. The values of correlation coefficient for the correlation between mortality to incidence ratios and human development index and current healthcare expenditure per gross domestic product were not correct.

A correction has been made to **Abstract**, *Results*, paragraph 1. This sentence previously stated:

“Also, HDI demonstrated weak significant correlations with HC ASIR (r= 0.249, p<0.01), ASMR (r= 0.185, p<0.05), and MIR (r= 0.347, p<0.001). Moreover, a weak significant correlation was also observed between CHE/GDP and MIR (r= 0.279, p<0.001).”

The corrected sentence appears below:

“Also, HDI demonstrated significant correlations with HC ASIR (r= 0.249, p<0.01), ASMR (r= 0.185, p<0.05), and MIR (r= -0.449, p<0.001). Moreover, a weak significant correlation was also observed between CHE/GDP and MIR (r= -0.295, p<0.001).”

In the published article, there was an error. The interpretations based on the correlations between mortality to incidence ratios and human development index and current healthcare expenditure per gross domestic product were not correct.

A correction has been made to **Discussion**, Paragraph 6. This sentence previously stated:

“On the other hand, analyzing the general correlations of developmental metrics with incidence rates, suggested a weak positive correlation between HDI and all metrics of ASIR, ASMR, and MIR, and also a weak positive correlation between CHE/GDP and MIR. However, despite our finding that the low HDI countries had the highest MIR values for HC, we observed a generally weak positive correlation between MIR and both metrics of HDI and CHE/GDP, which might be suggestive that the MIR is mostly influenced in low HDI countries, and the differences are less pronounced among countries with medium to very high HDI.”

The corrected sentence appears below:

“On the other hand, analyzing the general correlations of developmental metrics with incidence rates, suggested a positive correlation between HDI and ASIR and ASMR. Our findings showed that the low HDI countries had the highest MIR values for HC, and we observed a generally negative correlation between MIR and both metrics of HDI and CHE/GDP.”

The authors apologize for these errors and state that this does not change the scientific conclusions of the article in any way. The original article has been updated.

