# Supplementary material for: Global epidemiology and socioeconomic correlates of hypopharyngeal cancer in 2020 and its projection to 2040: findings from GLOBOCAN 2020
Source: Front Oncol. 2024 Sep 2;14:1398063. doi: 10.3389/fonc.2024.1398063 (PMC11402725; doi:10.3389/fonc.2024.1398063)
Supplement: Supplementary file 1 [file DataSheet1.docx]

**Supplementary Tables**

| **Table S1.** Male hypopharyngeal cancer incidence and mortality metrics in 2020 for different geographic and socioeconomic categories. | | | | | | | | | | | |
| --- | --- | --- | --- | --- | --- | --- | --- | --- | --- | --- | --- |
| **Location** | **Incidence** | | | | | **Mortality** | | | | | **MIR** |
|  | **Number** | **Uncertainty interval** | **Crude rate** | **ASIR** | **Cumulative risk (%)** | **Number** | **Uncertainty interval** | **Crude rate** | **ASMR** | **Cumulative risk (%)** |  |
| **World** | 70254 | 63472.7-77759.9 | 1.80 | 1.60 | 0.33 | 32303 | 28352.2-36804.3 | 0.82 | 0.72 | 0.17 | 0.46 |
| **WHO regions** | | | | | | | | | | | |
| **WHO Africa (AFRO)** | 1079 | 434.6-2678.8 | 0.19 | 0.37 | 0.07 | 795 | 281.6-2244.2 | 0.14 | 0.27 | 0.05 | 0.74 |
| **WHO Americas (PAHO)** | 4068 | 3596.1-4601.8 | 0.81 | 0.60 | 0.13 | 1466 | 1214.0-1770.3 | 0.29 | 0.21 | 0.05 | 0.36 |
| **WHO East Mediterranean (EMRO)** | 1964 | 1156.8-3334.6 | 0.52 | 0.72 | 0.18 | 928 | 527.4-1632.9 | 0.25 | 0.35 | 0.10 | 0.48 |
| **WHO Europe (EURO)** | 17189 | 14983.9-19718.6 | 3.80 | 2.40 | 0.41 | 8633 | 7241.7-10291.6 | 1.90 | 1.20 | 0.22 | 0.50 |
| **WHO South-East Asia (SEARO)** | 31315 | 28633.5-34247.7 | 3.00 | 3.20 | 0.73 | 13043 | 9960.1-17080.1 | 1.30 | 1.30 | 0.34 | 0.43 |
| **WHO Western Pacific (WPRO)** | 14613 | 10091.0-21161.5 | 1.50 | 0.97 | 0.23 | 7428 | 4171.3-13227.5 | 0.74 | 0.49 | 0.15 | 0.49 |
| **Continent** | | | | | | | | | | | |
| **Africa** | 1353 | 577.9-3167.8 | 0.20 | 0.36 | 0.08 | 965 | 366.7-2539.4 | 0.14 | 0.26 | 0.06 | 0.70 |
| **Asia** | 47903 | 43415.8-52853.9 | 2.00 | 1.80 | 0.40 | 21361 | 17498.2-26076.5 | 0.90 | 0.78 | 0.21 | 0.45 |
| **Europe** | 16727 | 15702.0-17818.9 | 4.60 | 2.70 | 0.45 | 8433 | 7551.4-9417.5 | 2.30 | 1.30 | 0.24 | 0.50 |
| **Latin America and the Caribbean** | 2054 | 1643.2-2567.5 | 0.64 | 0.58 | 0.14 | 914 | 787.7-1060.6 | 0.28 | 0.26 | 0.06 | 0.44 |
| **Northern America** | 2014 | 1919.2-2113.5 | 1.10 | 0.63 | 0.13 | 552 | 500.1-609.3 | 0.30 | 0.16 | 0.04 | 0.27 |
| **Oceania** | 203 | 163.5-252.1 | 0.95 | 0.65 | 0.16 | 78 | 52.4-116.2 | 0.37 | 0.23 | 0.07 | 0.39 |
| **World Bank income levels** | | | | | | | | | | | |
| **Low income** | 575 | 312.2-1059.1 | 0.19 | 0.36 | 0.07 | 393 | 198.5-778.0 | 0.13 | 0.24 | 0.05 | 0.68 |
| **Low middle income** | 36508 | 32787.5-40650.7 | 2.40 | 2.90 | 0.67 | 15901 | 11802.2-21423.2 | 1.00 | 1.30 | 0.32 | 0.42 |
| **Upper middle income** | 13964 | 12513.3-15582.9 | 0.95 | 0.73 | 0.15 | 7257 | 6083.7-8656.6 | 0.49 | 0.38 | 0.08 | 0.52 |
| **High income** | 19198 | 18043.9-20425.9 | 3.10 | 1.80 | 0.34 | 8751 | 7956.4-9624.9 | 1.40 | 0.74 | 0.18 | 0.45 |
| **HDI categories** | | | | | | | | | | | |
| **Low HDI** | 741 | 314.2-1747.6 | 0.15 | 0.30 | 0.06 | 546 | 209.6-1422.3 | 0.11 | 0.22 | 0.04 | 0.73 |
| **Medium HDI** | 34043 | 30895.5-37511.1 | 2.90 | 3.30 | 0.77 | 14461 | 10310.2-20282.9 | 1.20 | 1.40 | 0.37 | 0.41 |
| **High HDI** | 11477 | 10029.5-13133.4 | 0.78 | 0.61 | 0.13 | 6011 | 5053.5-7149.9 | 0.41 | 0.32 | 0.07 | 0.53 |
| **Very high HDI** | 23984 | 22574.4-25481.6 | 3.10 | 1.90 | 0.36 | 11284 | 10154.2-12539.5 | 1.50 | 0.83 | 0.19 | 0.48 |
| **ASIR:** Age-standardized incidence rate; **ASMR:** Age-standardized mortality rate; **MIR:** Mortality-to-incidence ratio; **WHO:** World Health Organization; **HDI:** Human Development Index. Rates are presented per 100000 population. | | | | | | | | | | | |

| **Table S2.** Male hypopharyngeal cancer incidence and mortality metrics for age groups. | | | | | | | |
| --- | --- | --- | --- | --- | --- | --- | --- |
| **Age group** | **Incidence** | | | **Mortality** | | | **MIR** |
|  | **Number** | **Crude rate** | **Cumulative risk (%)** | **Number** | **Crude rate** | **Cumulative risk (%)** |  |
| 0 to 9 | 35 | 0.01 | 0 | 13 | 0 | 0 | 0 |
| 10 to 19 | 83 | 0.01 | 0 | 51 | 0.01 | 0 | 1.00 |
| 20 to 29 | 391 | 0.05 | 0 | 173 | 0.03 | 0 | 0.60 |
| 30 to 39 | 1692 | 0.29 | 0 | 864 | 0.15 | 0 | 0.52 |
| 40 to 49 | 7890 | 1.60 | 0.02 | 3044 | 0.62 | 0.01 | 0.39 |
| 50 to 59 | 18369 | 4.40 | 0.04 | 7016 | 1.70 | 0.02 | 0.39 |
| 60 to 69 | 22943 | 8.00 | 0.08 | 10228 | 3.60 | 0.04 | 0.45 |
| 70+ | 18923 | 9.60 | 0.18 | 10914 | 5.50 | 0.11 | 0.57 |
| **MIR:** Mortality-to-incidence ratio | | | | | | | |

| **Table S3.** Female hypopharyngeal cancer incidence and mortality metrics in 2020 for different geographic and socioeconomic categories. | | | | | | | | | | | |
| --- | --- | --- | --- | --- | --- | --- | --- | --- | --- | --- | --- |
| **Location** | **Incidence** | | | | | **Mortality** | | | | | **MIR** |
|  | **Number** | **Uncertainty interval** | **Crude rate** | **ASIR** | **Cumulative risk (%)** | **Number** | **Uncertainty interval** | **Crude rate** | **ASMR** | **Cumulative risk (%)** |  |
| **World** | 14000 | 10847.2-18069.2 | 0.36 | 0.29 | 0.05 | 6296 | 4653.8-8517.7 | 0.16 | 0.13 | 0.03 | 0.44 |
| **WHO regions** | | | | | | | | | | | |
| **WHO Africa (AFRO)** | 443 | 104.3-1881.7 | 0.08 | 0.14 | 0.04 | 324 | 62.2-1687.7 | 0.06 | 0.10 | 0.03 | 0.75 |
| **WHO Americas (PAHO)** | 824 | 624.7-1086.9 | 0.16 | 0.11 | 0.02 | 274 | 176.1-426.3 | 0.05 | 0.03 | 0.01 | 0.31 |
| **WHO East Mediterranean (EMRO)** | 1058 | 576.2-1942.7 | 0.30 | 0.36 | 0.06 | 477 | 198.3-1147.7 | 0.13 | 0.16 | 0.03 | 0.43 |
| **WHO Europe (EURO)** | 2541 | 1719.5-3754.9 | 0.53 | 0.30 | 0.05 | 1088 | 638.0-1855.4 | 0.23 | 0.12 | 0.02 | 0.43 |
| **WHO South-East Asia (SEARO)** | 7633 | 6313.9-9227.7 | 0.77 | 0.73 | 0.14 | 3293 | 1658.1-6539.9 | 0.33 | 0.31 | 0.07 | 0.43 |
| **WHO Western Pacific (WPRO)** | 1500 | 311.5-7224.0 | 0.16 | 0.09 | 0.02 | 840 | 123.7-5704.2 | 0.09 | 0.05 | 0.01 | 0.56 |
| **Continent** | | | | | | | | | | | |
| **Africa** | 712 | 234.3-2164.0 | 0.11 | 0.17 | 0.04 | 474 | 133.9-1678.1 | 0.07 | 0.11 | 0.03 | 0.64 |
| **Asia** | 10155 | 7882.1-13083.4 | 0.45 | 0.36 | 0.07 | 4545 | 2536.5-8143.9 | 0.20 | 0.16 | 0.03 | 0.44 |
| **Europe** | 2269 | 1897.9-2712.6 | 0.59 | 0.31 | 0.05 | 985 | 728.2-1332.3 | 0.25 | 0.12 | 0.02 | 0.42 |
| **Latin America and the Caribbean** | 376 | 219.1-645.2 | 0.11 | 0.09 | 0.02 | 162 | 109.9-238.8 | 0.05 | 0.04 | 0.01 | 0.45 |
| **Northern America** | 448 | 404.2-496.6 | 0.24 | 0.13 | 0.03 | 112 | 90.0-139.4 | 0.06 | 0.03 | 0.01 | 0.25 |
| **Oceania** | 40 | 22.6-70.8 | 0.19 | 0.13 | 0.03 | 18 | 7.8-41.6 | 0.08 | 0.05 | 0.01 | 0.42 |
| **World Bank income levels** | | | | | | | | | | | |
| **Low income** | 284 | 104.8-769.6 | 0.09 | 0.15 | 0.04 | 190 | 62.3-579.2 | 0.06 | 0.10 | 0.03 | 0.67 |
| **Low middle income** | 9170 | 7301.8-11516.3 | 0.62 | 0.66 | 0.13 | 4103 | 1984.9-8481.5 | 0.28 | 0.29 | 0.06 | 0.45 |
| **Upper middle income** | 1787 | 1268.2-2518.1 | 0.12 | 0.08 | 0.02 | 947 | 562.5-1594.3 | 0.07 | 0.04 | 0.01 | 0.58 |
| **High income** | 2759 | 2328.6-3268.9 | 0.45 | 0.23 | 0.04 | 1056 | 820.6-1359.0 | 0.17 | 0.08 | 0.02 | 0.38 |
| **HDI categories** | | | | | | | | | | | |
| **Low HDI** | 376 | 106.9-1322.0 | 0.08 | 0.14 | 0.04 | 267 | 65.7-1085.9 | 0.05 | 0.10 | 0.03 | 0.63 |
| **Medium HDI** | 8702 | 7112.7-10646.4 | 0.77 | 0.80 | 0.16 | 3835 | 1790.3-8214.7 | 0.34 | 0.35 | 0.08 | 0.44 |
| **High HDI** | 1547 | 989.1-2419.7 | 0.11 | 0.08 | 0.02 | 819 | 482.6-1389.8 | 0.06 | 0.04 | 0.01 | 0.55 |
| **Very high HDI** | 3375 | 2851.8-3994.1 | 0.43 | 0.24 | 0.04 | 1375 | 1039.2-1819.3 | 0.17 | 0.09 | 0.02 | 0.40 |
| **ASIR:** Age-standardized incidence rate; **ASMR:** Age-standardized mortality rate; **MIR:** Mortality-to-incidence ratio; **WHO:** World Health Organization; **HDI:** Human Development Index. Rates are presented per 100000 population. | | | | | | | | | | | |

| **Table S4.** Female hypopharyngeal cancer incidence and mortality metrics for age groups. | | | | | | | |
| --- | --- | --- | --- | --- | --- | --- | --- |
| **Age group** | **Incidence** | | | **Mortality** | | | **MIR** |
|  | **Number** | **Crude rate** | **Cumulative risk (%)** | **Number** | **Crude rate** | **Cumulative risk (%)** |  |
| 0 to 9 | 14 | 0 | 0 | 5 | 0 | 0 | 0 |
| 10 to 19 | 26 | 0 | 0 | 10 | 0 | 0 | 0 |
| 20 to 29 | 233 | 0.04 | 0 | 118 | 0.02 | 0 | 0.50 |
| 30 to 39 | 1038 | 0.18 | 0 | 593 | 0.11 | 0 | 0.61 |
| 40 to 49 | 2006 | 0.42 | 0 | 839 | 0.17 | 0 | 0.40 |
| 50 to 59 | 3399 | 0.81 | 0.01 | 1155 | 0.28 | 0 | 0.35 |
| 60 to 69 | 3778 | 1.20 | 0.01 | 1580 | 0.52 | 0.01 | 0.43 |
| 70+ | 3506 | 1.30 | 0.03 | 1996 | 0.77 | 0.02 | 0.59 |
| **MIR:** Mortality-to-incidence ratio. | | | | | | | |

**Supplementary Figures**

**
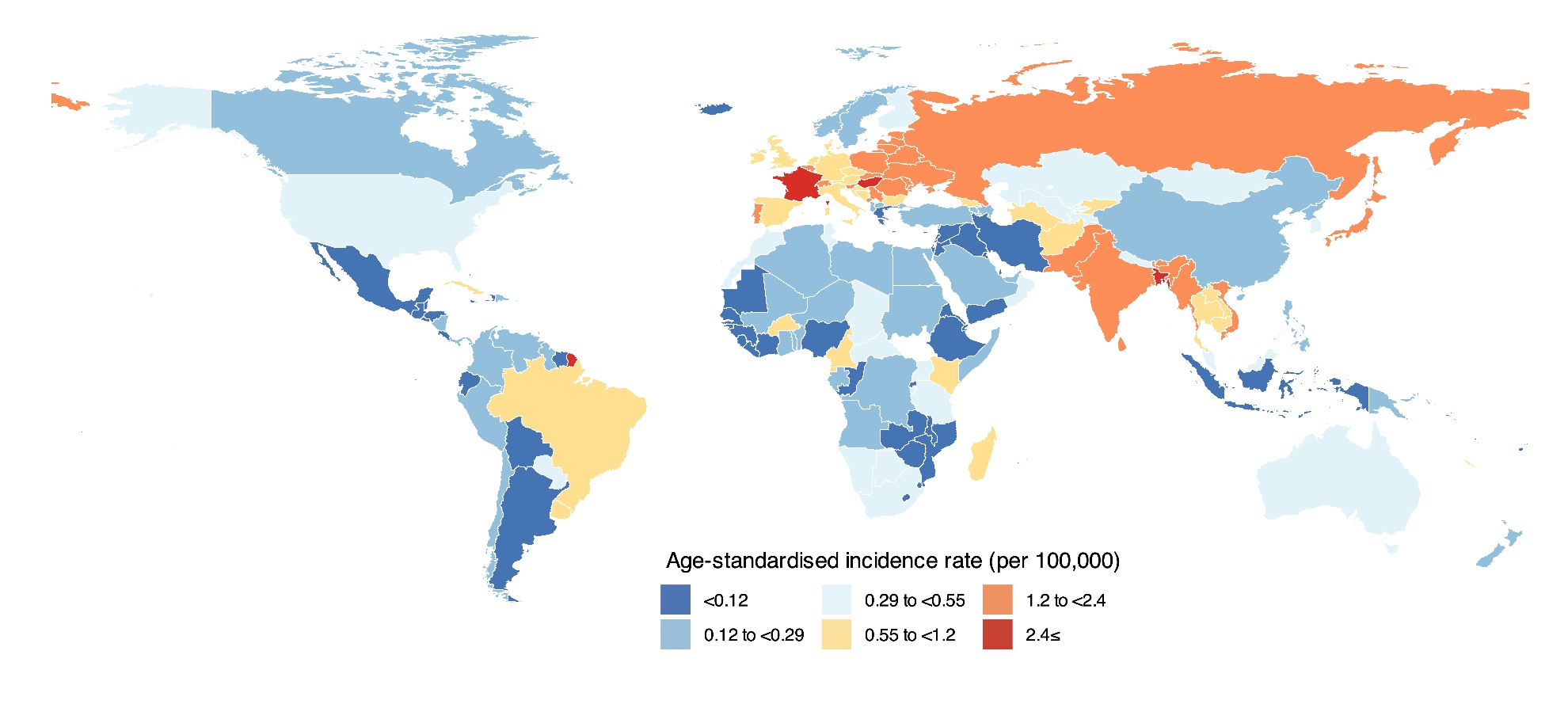
**

**Figure S1.** Global distribution of male hypopharyngeal cancer based on age-standardized incidence rate.


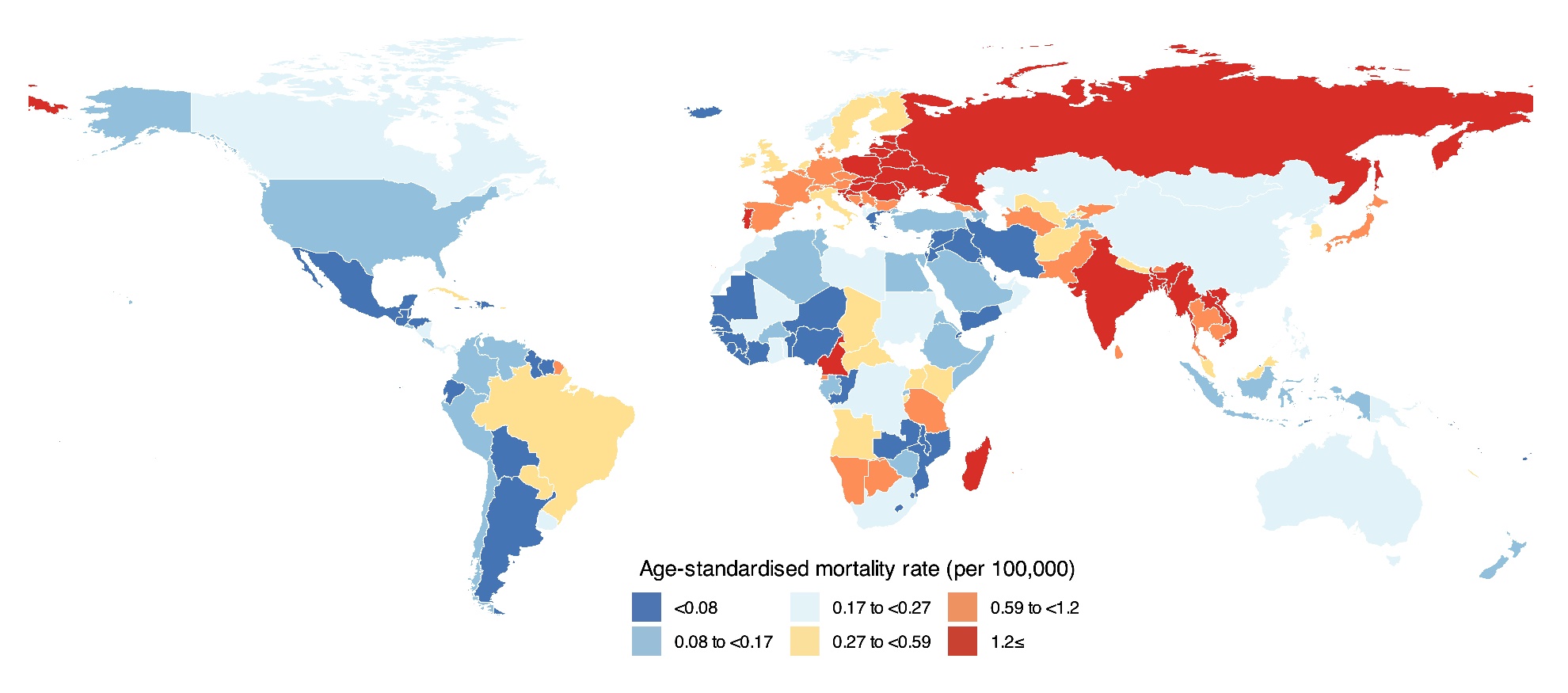


**Figure S2.** Global distribution of male hypopharyngeal cancer based on age-standardized mortality rate.


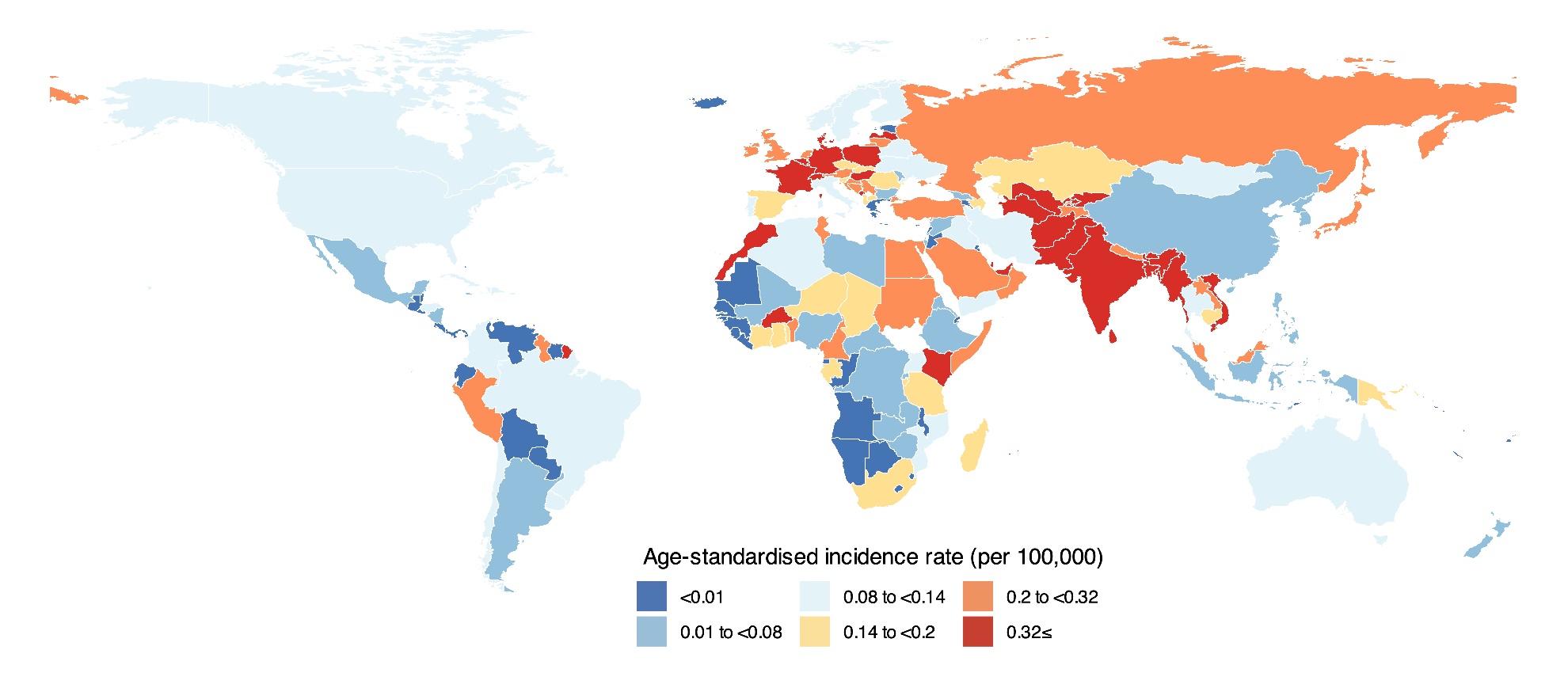


**Figure S3.** Global distribution of female hypopharyngeal cancer based on age-standardized incidence rate.


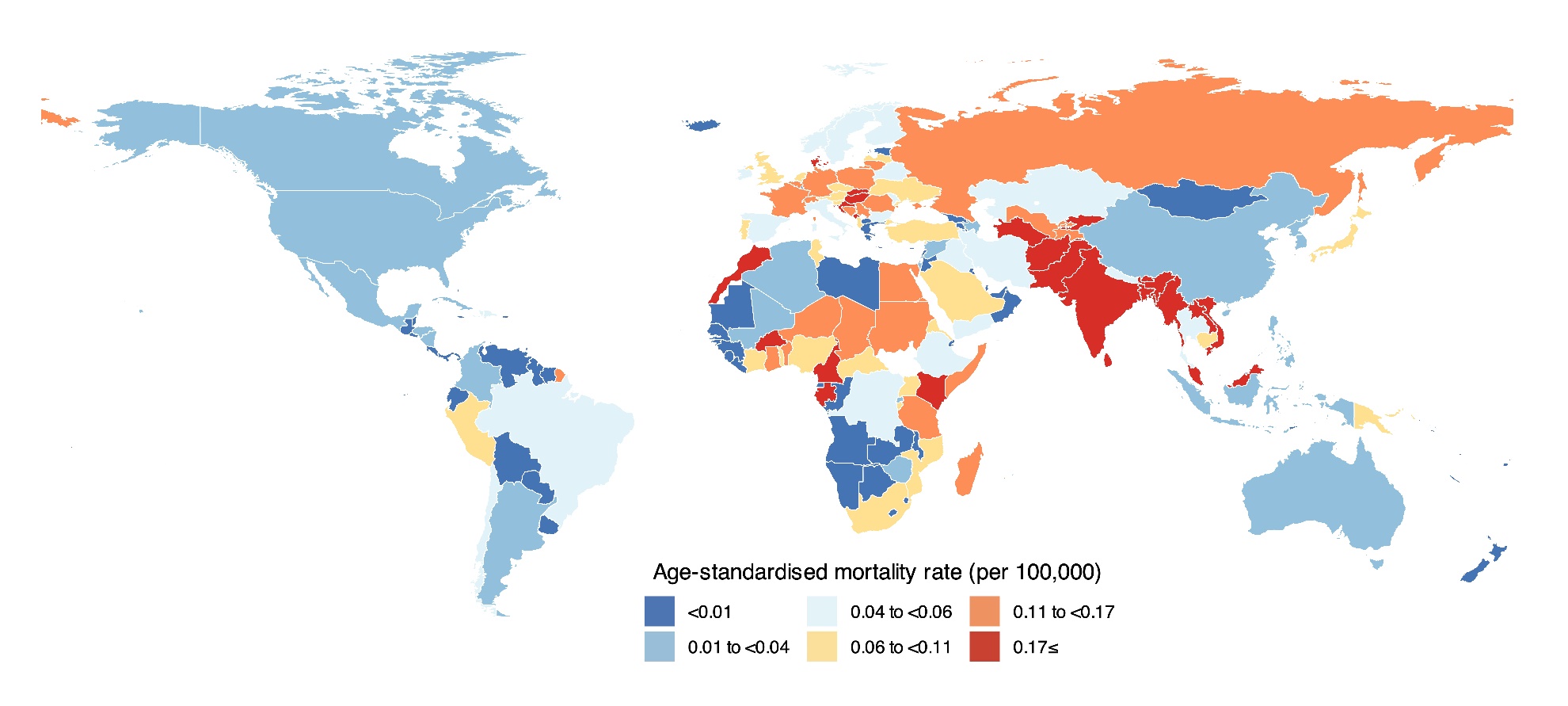


**Figure S4.** Global distribution of female hypopharyngeal cancer based on age-standardized mortality rate.
